# Author Correction: Targeting QKI-7 in vivo restores endothelial cell function in diabetes

**DOI:** 10.1038/s41467-020-18712-1

**Published:** 2020-09-16

**Authors:** Chunbo Yang, Magdalini Eleftheriadou, Sophia Kelaini, Thomas Morrison, Marta Vilà González, Rachel Caines, Nicola Edwards, Andrew Yacoub, Kevin Edgar, Arya Moez, Aleksandar Ivetic, Anna Zampetaki, Lingfang Zeng, Fiona L. Wilkinson, Noemi Lois, Alan W. Stitt, David J. Grieve, Andriana Margariti

**Affiliations:** 1 The Wellcome-Wolfson Institute of Experimental Medicine, Belfast, BT9 7BL UK; 2 Centre for Bioscience in the Department of Life Sciences, Faculty of Science and Engineering, Manchester Metropolitan University, Manchester, M15GD UK; 3 School of Cardiovascular Medicine and Sciences, BHF Centre of Research Excellence, King’s College London, The James Black Centre, 125 Coldharbour Lane, London, SE5 9NU UK

**Keywords:** Gene regulation, Induced pluripotent stem cells, Diabetes complications

Correction to: *Nature Communications* 10.1038/s41467-020-17468-y, published online 30 July 2020.

The original version of this Article’s Acknowledgement section incorrectly omitted:

The support of the Belfast Association for the Blind.

This has now been corrected in both the PDF and HTML versions of the Article.

